# MAC-spinal meningioma score: A proposal for a quick-to-use scoring sheet of the MIB-1 index in sporadic spinal meningiomas

**DOI:** 10.3389/fonc.2022.966581

**Published:** 2022-08-26

**Authors:** Johannes Wach, Motaz Hamed, Tim Lampmann, Ági Güresir, Frederic Carsten Schmeel, Albert J. Becker, Ulrich Herrlinger, Hartmut Vatter, Erdem Güresir

**Affiliations:** ^1^ Department of Neurosurgery, University Hospital Bonn, Bonn, Germany; ^2^ Department of Neuroradiology, University Hospital Bonn, Bonn, Germany; ^3^ Department of Neuropathology, University Hospital Bonn, Bonn, Germany; ^4^ Department of Neurology, Section of Neuro-Oncology, University Hospital Bonn, Bonn, Germany

**Keywords:** MIB-1 (Ki-67 labeling) index, score, spinal meningioma, proliferation, clinical implications

## Abstract

**Objective:**

MIB-1 index is an important predictor of meningioma progression. However, MIB-1 index is not available in the preoperative tailored medical decision-making process. A preoperative scoring sheet independently estimating MIB-1 indices in spinal meningioma (SM) patients has not been investigated so far.

**Methods:**

Between 2000 and 2020, 128 patients with clinical data, tumor imaging data, inflammatory laboratory (plasma fibrinogen, serum C-reactive protein) data, and neuropathological reports (MIB-1, mitotic count, CD68 staining) underwent surgery for spinal WHO grade 1 and 2 meningioma.

**Results:**

An optimal MIB-1 index cut-off value (≥5/<5) predicting recurrence was calculated by ROC curve analysis (AUC: 0.83; 95%CI: 0.71-0.96). An increased MIB-1 index (≥5%) was observed in 55 patients (43.0%) and multivariable analysis revealed significant associations with baseline Modified McCormick Scale ≥2, age ≥65, and absence of calcification. A four-point scoring sheet (MAC-Spinal Meningioma) based on **M**odified McCormick, **A**ge, and **C**alcification facilitates prediction of the MIB-1 index (sensitivity 71.1%, specificity 60.0%). Among those patients with a preoperative MAC-Meningioma Score ≥3, the probability of a MIB-1 index ≥5% was 81.3%.

**Conclusion:**

This novel score (MAC-Spinal Meningioma) supports the preoperative estimation of an increased MIB-1 index, which might support preoperative patient-surgeon consultation, surgical decision making and enable a tailored follow-up schedule or an individual watch-and-wait strategy.

## Introduction

Spinal meningiomas (SM) account for only 12% of all anatomic types of meningiomas (1–5). Spinal meningiomas are predominantly benign and slowly growing WHO grade 1 tumors. However, higher WHO grades are also reported and the frequency of them ranges between 1.5 and 8.5% (6–10). Gross total microsurgical removal is the treatment of choice for those meningiomas (11, 12). The majority of patients who underwent surgical SM resection improve regarding neurological functioning (10, 13). However, patients ≥ 66 years were found to have significant poorer recovery. The tumor recurrence rate in spinal meningiomas range between 1.3 and 13% (4, 6, 14–20). In addition to the extent of resection, male sex, dural tail sign, younger age, tumor size, foraminal location and en plaque lesions were suggested as predictors of tumor recurrence after spinal meningioma surgery (21, 22)

Increased proliferative activity of tumor cells is an established mechanism of oncogenesis (23, 24). The Molecular Immunology Borstel 1 (MIB-1) index is a widespread immunohistochemical method to detect nuclear structures which are exclusively visible in proliferating cells. The Ki-67 antigen is detectable in the nuclei of cells which are in G1, S, and G2 phases of the cell division cycle. Hence, this method enables a calculation of the growing fraction of a meningioma tissue (25–27). Furthermore, several investigations and meta-analyses revealed that the MIB-1 index is an independent risk factor for tumor progression in meningiomas (28–31). Tailored preoperative evaluation, accurate communication about the aims of surgery, and maximum safe surgery with preservation of neurological functioning are of paramount importance. However, MIB-1 index is not available as a basis for a detailed tailored consultation in the preoperative surgical decision-making and surgeon-patient conversation. In a previous institutional series, we identified that the MIB-1 labeling indices in spinal meningiomas are significantly lower compared to the cranial meningiomas. Hence, sufficient predictors of MIB-1 labeling indices in spinal meningiomas have to be investigated separately from cranial meningiomas (32).

The present study investigates our patient cohort of sporadic spinal WHO grade 1 and 2 meningiomas regarding potential clinical characteristics, laboratory inflammatory markers, and imaging features as predictors of an elevated MIB-1 index.

## Methods

### Patient population

This investigation reviewed 130 consecutive SM patients who underwent surgery between 2000 and 2020. The aim of the present single-center series is focused on the investigation of SMs located below the craniocervical junction. Patients with craniocervical meningiomas (occipital bone, C1, C2), patients with anterior foramen magnum meningiomas, a recurrent meningioma after radiotherapy, and neurofibromatosis type 2 patients were excluded because of their different clinical symptoms, neuropathology, and treatment strategies (33–36). Patients without neuropathological reports regarding the MIB-1 index were excluded. One-hundred-twenty-eight patients were included in the final study cohort.

### Data recording and radiological features

**Figure 1:**
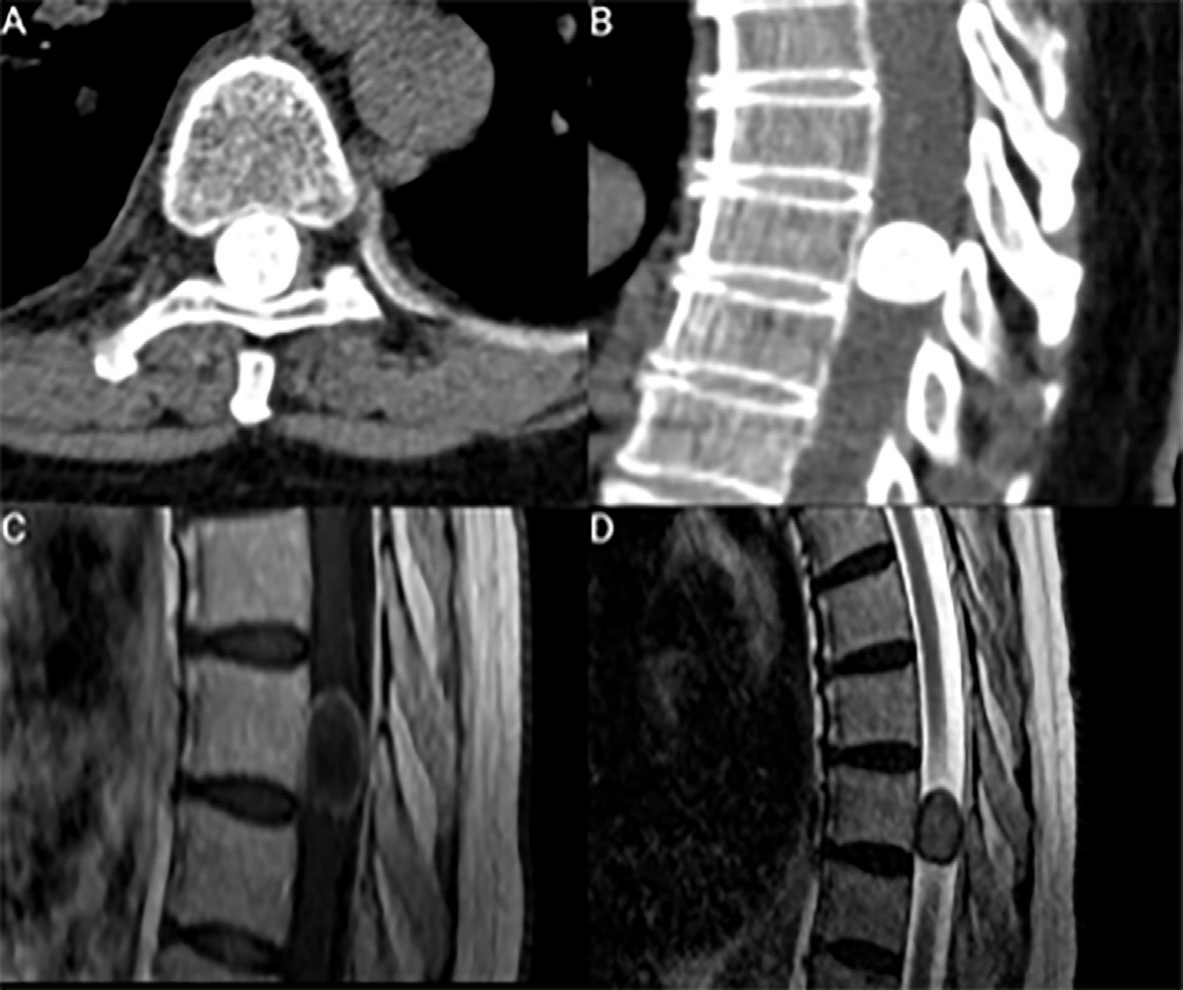
**(A, B)** Axial and sagittal CT scans showing a representative case with a gross calcification of a thoracic spinal meningioma. **(C, D)** represent sagittal T1-weighted Gd-enhanced and T2-weighted MR-images. **(C)** shows a heterogeneous ring-enhancing lesion because of the gross calcification.

Clinical data such as age, sex, comorbidities, Karnofsky Performance Status, body mass index (BMI), length of stay (in days) and the American society of anesthesiologists physical status classification system (ASA) were recorded in a computerized database (SPSS, version 27 for Windows, IBM Corp., Armonk, NY). Preoperative neurological examination was performed by institutional neurosurgeons and the modified McCormick Scale (MMS) was used to display neurological functioning and ambulatory ability (37). MMS was dichotomized into “good” (I&II) and “poor” (III-V) functioning as previously described (13, 38). Preoperative magnetic resonance imaging (MRI) was conducted within 72 hours prior to surgical treatment. Furthermore, all patients preoperatively underwent CT-scans of the spine segment under investigation. Calcification was confirmed by CT scans representing focal or diffuse hyperdense gross calcifications (39). On MRI, calcification resulted in signal intensity decrease on both T1- and T2-weighted MR images and a more heterogeneous Gd-enhancement compared to the typically encountered MR-imaging characteristics (e.g., homogeneous Gd-enhancement) of meningioma (40) (**Figure 1**). First postoperative MR imaging was scheduled at 3-months after surgery and further appointments for MRI were scheduled on an annual basis (41). Spinal meningioma recurrence was defined as a visible meningioma progression on follow-up MRI at least one year after surgery (42). T2-weighted images showing high signal intensity changes of the spinal cord were interpreted as myelomalacia (43).

### Surgical workflow

Surgery was indicated in case of local back pain combined with absence of competing spinal pathologies, neurological deficits, and compression of the spinal cord. Surgical strategy was dependent on the site of dural attachment of the meningioma, meningioma size, as well as the involved spinal segment of the SM. Hemilaminectomy or laminoplasty was performed in order to preserve functional stability of the spine. Dentate ligament was resected if the SM had a ventral dural attachment. Dural closure and reconstruction was performed with continuous silk sutures and additionally sealed with TachoSil^®^ (Fibrin Sealant Patch) if deemed necessary. Further surgical workflow was as previously described (13).

### Histopathology

Neuropathological classification is in line with 2021 WHO criteria (11). Classification and grading of spinal meningiomas did not undergo substantial revision in 2021. Immunohistochemical staining was performed in a similar workflow as described before for paraffin-embedded biopsy tissue specimens (44, 45). The MIB-1 labeling index was determined using the following antibody: anti-Ki67 (Clone Ki-67P, dilution 1:1000, DAKO, Glostrup, Denmark) (24). Visualization was performed with diaminobenzidine, and histopathological investigation was conducted by expert neuropathologists, including A.J.B. The MIB-1 index was analyzed in randomly selected high-power microscopic fields. The amounts of stained and unstained nuclei in the meningioma cells were determined. Further neuropathological examinations were as previously described (24, 46).

### Statistical analysis

Data were recorded and analyzed using SPSS for Windows (version 27.0; IBM Corp, Armonk, NY, USA). Receiver-operating characteristic curves (ROC) were created to investigate the diagnostic performance of MIB-1 labeling index in the prediction of a spinal meningioma recurrence. Cut-off point for the MIB-1 labeling index was set based on the ROC analysis. Kaplan-Meier charts of progression-free survival (PFS) stratified by MIB-1 labeling indices as well as extent of resection according to the Simpson grading were also calculated. Statistical results of the log-rank test are reported. Normally distributed data are presented as the mean with the standard deviation (SD). Preoperative demographics, clinical data, imaging characteristics, and inflammatory laboratory markers were compared between patients with a normal and those with an elevated MIB-1 labeling index using Pearson´s χ2 test (two-sided) for categorical data and independent *t*-test for continuous data. Further ROC curves were constructed for age and MMS. The area under the ROC curve (AUC) were investigated, and cut-off thresholds for the continuous variables (age & MMS) were set using the ROC analyses. Multivariable binary logistic regression analysis was performed to identify predictive variables of an elevated MIB-1 labeling index. A *p*-value threshold of <0.10 in the univariable analysis was set regarding the inclusion of variables in the multivariable binary logistic regression analysis. Furthermore, sex was also included in the multivariable analysis of factors being associated with an increased MIB-1 labeling index because of the known strong evidence suggesting male sex as a predictor of elevated MIB-1 labeling indices in cranial meningiomas (24, 47, 48). Wald test was used for the analysis of dichotomized variables. A *p*-value of <0.05 was defined as statistically significant. Significant predictors of the multivariable analysis were included in a 4-point scoring sheet predicting an increased MIB-1 labeling index.

## Results

### Patient characteristics

One hundred and twenty-eight patients fulfilled the inclusion criteria and were surgically treated for SM at the institutional department. Median age was 68 years (IQR 57-75), and the present investigation included 98 females (76.6%) and 30 males (23.4%; female/male ratio 3.27:1). Median baseline Karnofsky performance scale (KPS was 80 (IQR 70-90). Tumors were predominantly located in the thoracic spine. Tumor classification according to the WHO classification criteria included 119 patients with WHO grade 1 (93.0%) and 9 patients with grade 2 (7.0%). Further characteristics are summarized in **Table 1**. Regarding histopathological type among WHO grade 1 SMs, psammomatous meningioma (68/119; 57.1%) was the most common subtype. Transitional, meningothelial, fibroblastic, and angiomatous subtypes were observed in 25 (25/119; 21.0%), 18 (18/119; 15.1%), 6 (6/119; 5.0%), and 2 (2/119; 1.7%) WHO grade 1 SMs patients, respectively. Atypical meningioma was observed in all cases among the WHO grade 2 SMs.

**Table 1:** Patient characteristics (n = 128).

Median age (IQR) (in y)	68 (57-75)
Sex	
Female	98 (76.6%)
Male	30 (23.4%)
Median preoperative KPS (IQR)	80 (70-90)
Tumor location
Cervical	32 (25.0%)
Thoracic	94 (73.4%)
Lumbar	2 (1.6%)
Simpson grade
Simpson grade I&II	123 (96.1%)
Simpson grade ≥ III	5 (3.9%)
WHO grade
WHO grade 1	119 (93.0%)
WHO grade 2	9 (7.0%)

### MIB-1 labeling index in the prediction of recurrent spinal meningioma

**Figure 2:**
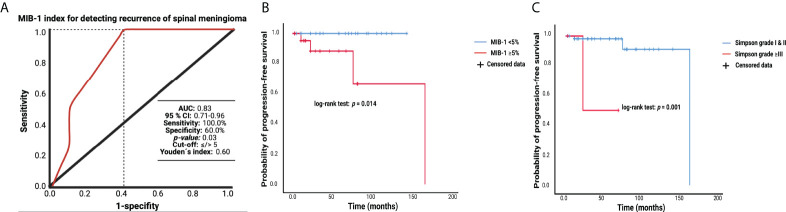
**(A)** Receiver-operating characteristic curve showing the MIB-1 labeling index in the prediction of progression of sporadic spinal meningiomas. The dashed line marks the identified optimum cut-off value. **(B)** Kaplan-Meier analysis of tumor progression probability stratified by “MIB-1 ≥ 5%” (red line) and “MIB-1 < 5” (blue line). Vertical dashes represent censored data (constituting for progression-free at last follow-up) within the PFS curves. The time axis is right-censored at 200 months. **(C)** Kaplan-Meier charts of tumor progression probability stratified by “Simpson grade ≥III” (red line) and “Simpson grade I & II” (blue line). Vertical dashes represent censored data (constituting for progression-free at last follow-up) within the PFS curves. The time axis is right-censored at 200 months.

The MIB-1 index was available in all patients of the entire cohort. The median MIB-1 labeling index was 4.0 (IQR 3.0-5.0). A ROC curve was created, and the AUC of the MIB-1 labeling index in the diagnostic performance regarding SM recurrence was calculated. The AUC of the MIB-1 labeling index in the prediction of SM recurrence was 0.83 (95% CI: 0.71-0.96, *p* = 0.03). Sensitivity and specificity of the MIB-1 labeling index for the prediction of a recurrent SM were 100.0% and 60.0%, respectively (Youden´s index: 0.60), with a threshold of ≥5%. **Figure 2A** displays the ROC curve and summarizes the results of the statistical analysis. Median (range) and mean time of imaging follow-up (*n* = 88) were 14.0 (3.0-169.0) and 33.70 months, respectively. Analysis of PFS was performed in 88 (69.0%) of the 128 patients. Four recurrent SMs were detected in the group of patients with a MIB-1 labeling index ≥ 5%, whereas no recurrent SM was observed in the group of patients with a MIB-1 labeling index < 5%. **Figure 2B** displays the Kaplan-Meier curves of the MIB-1 labeling index groups (<5/≥5%). Furthermore, extent of resection according to the Simpson grading system was analyzed with regard to the probability of progression-free survival. Mean time to tumor progression in SM patients who underwent a Simpson grade I or II resection was 159.7 (95% CI: 143.5 – 175.9) months, and in those patients who underwent a Simpson grade ≥III resection it was 48.0 (95% CI: 14.7 – 81.3) months, respectively (log-rank test: *p* = 0.001). **Figure 2C** illustrates the Kaplan-Meier chart of progression-free survival stratified by Simpson grade.

### Association between the MIB-1 labeling index and clinical, imaging, and laboratory features

Fifty-five patients had a MIB-1 labeling index of ≥ 5%, and 73 patients had a MIB-1 labeling index of <5%. Patients with an elevated MIB-1 labeling index were significantly older compared to patients with a lower MIB-1 labeling index. Patients with a MIB-1 labeling index of ≥ 5% had also a significantly higher MMS at presentation (2.3 +/- 1.2 vs. 1.8 +/- 1.0; *p* = 0.008). Furthermore, patients with a lower MIB-1 labeling index (<5%) had significantly more often a calcification of the SM compared to patients with an elevated MIB-1 labeling index. Extent of resection was also homogeneously distributed among the SM patients with normal (<5%) MIB-1 labeling index or increased MIB-1 labeling index (≥5%). Among the patients with a normal MIB-1 labeling index (<5%, *n* = 73), 71 patients (71/73; 97.3%) underwent either a Simpson grade I or II resection, whereas 52 patients (52/55; 94.5%) of those with an increased MIB-1 labeling index (≥5%, *n* = 55) underwent either a Simpson grade I or II resection (Fisher´s exact test (two-sided): *p* = 0.65). Further clinical, imaging, and laboratory characteristics are detailed in **Table 2**.

**Table 2:** Baseline clinical, imaging and laboratory characteristics in spinal meningioma patients with a normal and increased MIB-I labeling index (*n* = 128).

Variable	MIB-I <5 % (*n*=73)	MIB-I ≥5 % (*n*=55)	*p*-value
Age (mean +/- SD))	62.7 +/- 14.0	67.8 +/- 11.0	**0.03**
Sex female male	56 (76.7%) 17(23.3%)	42 (76.4%) 13 (23.6%)	0.99
KPS (mean +/- SD)	81.6 +/- 11.1	82.0 +/- 11.0	0.86
Modified McCormick Scale (mean +/- SD)	1.8 +/- 1.0	2.3 +/- 1.2	**0.008**
Diabetes Present Not present	10 (13.7%) 63 (86.3%)	10 (18.2%) 45 (81.8%)	0.62
Smoking Present Not present	12 (16.4%) 61 (83.6%)	9 (16.4%) 46 (83.6%)	0.99
ASA intake Present Not present	8 (11.0%) 65 (89.0%)	10 (18.2%) 45 (81.8%)	0.36
Dexamethasone intake Present Not present	11 (15.1%) 62 (84.9%)	8 (14.5%) 47 (85.5%)	0.93
Location Cervical Thoracic & lumbar	20 (27.4%) 53 (72.6%)	12 (21.8%) 43 (78.2%)	0.54
Calcification Present Absent	10 (13.7%) 63 (86.3%)	1 (1.8%) 54 (98.2%)	**0.02**
Cysts Present Absent	2 (2.7%) 71 (97.3%)	0 (0.0%) 55 (100.0%)	0.51
Dural tail sign Present Absent	9 (12.3%) 64 (87.7%)	14 (25.5%) 41 (74.5%)	0.07
Dural attachment (one patient was excluded due to selective arachnoid attachment) Ventral Lateral Dorsal	19 (26.0%) 27 (37.0%) 27 (37.0%)	19 (35.2%) 24 (44.4%) 11 (20.4%)	0.13
Involved spinal segments ≤ 2 > 2	67 (91.8%) 6 (8.2%)	48 (87.3%) 7 (12.7%)	0.56
Myelomalacia (T2-weighted MR-image) Present Absent	39 (53.4%) 34 (46.6%)	28 (50.9%) 27 (49.1%)	0.86
Plasma Fibrinogen (mean +/- SD)	3.2 +/- 0.6	3.5 +/- 0.9	0.25
Serum C-reactive protein (mean +/- SD)	4.8 +/- 8.7	5.2 +/- 7.6	0.77
White blood cell count (mean +/- SD)	8.6 +/- 3.9	8.7 +/- 4.3	0.95
Simpson grade I & II ≥ III	71 (97.3%) 2 (2.7%)	52 (94.5%) 3 (5.5%)	0.65

Bold values represent statistically significant results (p<0.05).

**Figure 3:**
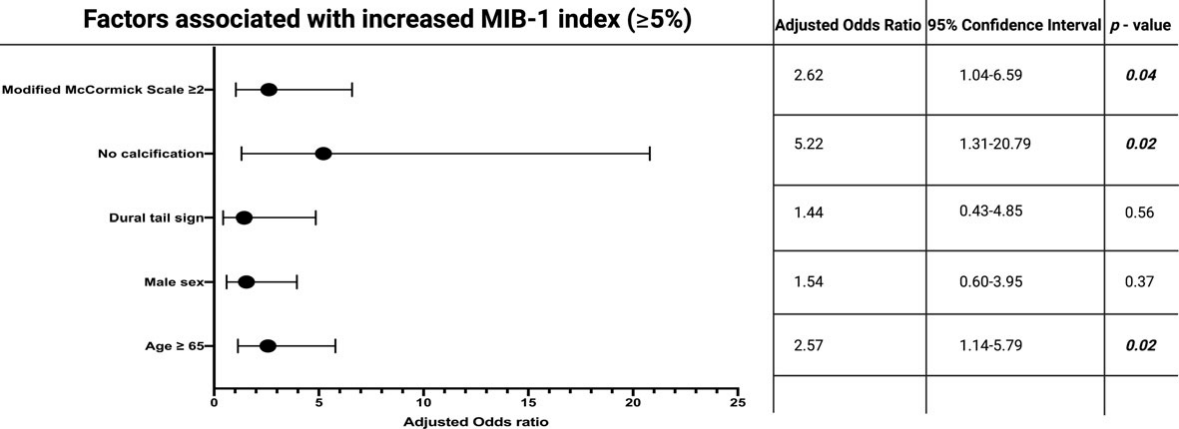
Forest plots from multivariable binary logistic regression analysis: Modified McCormick scale ≥ 2 at presentation, age ≥ 65 years at diagnosis, and absence of calcification are independent predictors of increased MIB-1 labeling index. Black circles indicate the adjusted odds ratio of each variable and the lines represent the corresponding 95% confidence interval. *P*-values in bold and italics display statistically significant results.

ROC curves were created, and the AUCs of age and baseline MMS in the prediction of an elevated MIB-1 labeling index (≥5%) were created. The AUCs for age and baseline MMS were 0.61 (95% CI: 0.51-0.72, *p =* 0.04) and 0.64 (95% CI: 0.54-0.74, *p =* 0.01). Optimum cut-off values for age and baseline MMS were identified at ≥65/<65 and ≥2/<2. The sensitivity and specificity of age at diagnosis for predicting a MIB-1 labeling index of ≥5% were 70.5% and 50.0%, respectively. Moreover, the sensitivity and specificity of baseline MMS for the prediction of an increased MIB-1 labeling index (≥5%) were 69.3% and 52.1%. Multivariable binary logistic regression analysis with consideration of MMS, calcification, dural tail sign, sex, and age was performed. The multivariable analysis found that MMS ≥ 2, age ≥ 65, and the absence of calcification were significantly associated with a MIB-1 labeling index of ≥ 5%. **Figure 3** displays the results of the multivariable binary logistic regression analysis.

### Predictive scoring sheet

**Figure 4:**
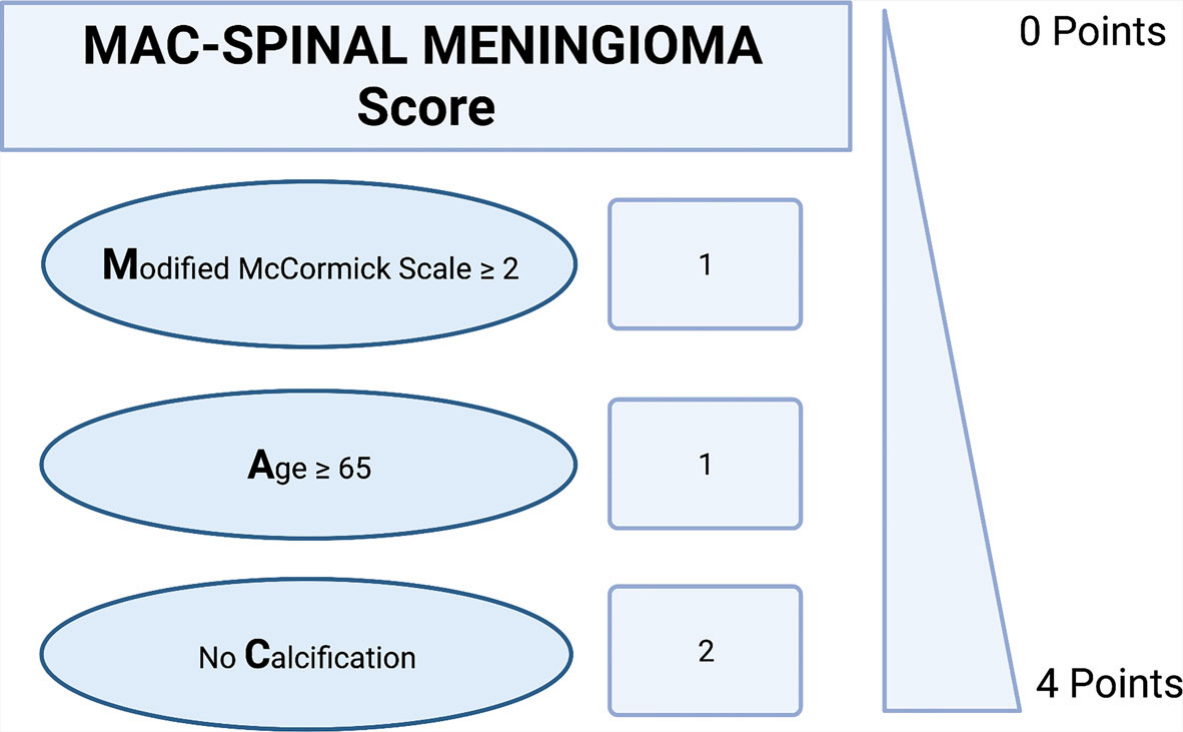
A proposal for a novel clinical scoring sheet to preoperatively estimate the risk of an increased MIB-1 labeling index (≥5%). An additive score of ≥ 3 points implies a probability of 81.3% for having an increased proliferative activity.

**Figure 5:**
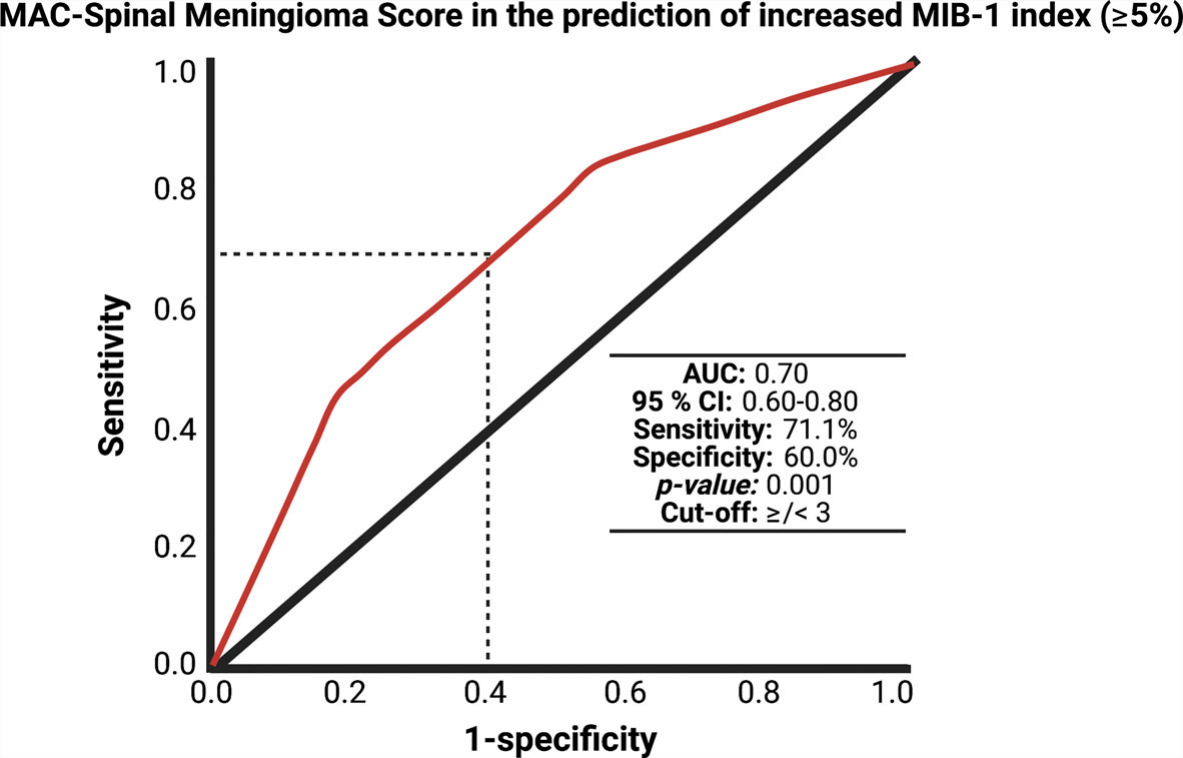
Receiver-operating characteristic curve demonstrating the MAC-Spinal Meningioma Score in the prediction of increased MIB-1 labeling index of sporadic spinal meningiomas. The dashed line marks the identified optimum cut-off value of the MAC-Spinal Meningioma score in the prediction of an increased MIB-1 labeling index (≥5%).

Hence, we created and investigated a proposal for a predictive scoring system of an increased MIB-1 labeling index in sporadic spinal meningioma. The present proposal was created with the following objectives: (1) to feasibly estimate the MIB-1 labeling index using easily determinable preoperative variables and (2) to quick-to-use in the clinical care for SM patients. These objectives resulted in the following point distribution system for a novel scoring sheet, which we called the “MAC-Spinal Meningioma” score, ranging from 0 to 4 points (**Figure 4**): Baseline Modified McCormick Scale ≥ 2 (1 point); age ≥ 65 years at diagnosis (1 point); absence of calcification (2 points). In the present study, the mean score in patients with a MIB-1 labeling index of ≥ 5% was 3.2 (SD = 0.78), and it was 2.67 (SD = 0.81) in patients with a MIB-1 labeling index of <5%, respectively (*p* < 0.001). The AUC for the MAC-Spinal Meningioma score in predicting an increased MIB-1 labeling index (≥5%) was 0.70 (95% CI: 0.60-0.80, *p* = 0.001). Using a cut-off value of 3 points, the score yields a sensitivity of 71.1%, a specificity of 60.0%, a positive predictive value of 81.3%, and a negative predictive value of 45.8%. **Figure 5** shows the ROC curve and the results of the statistical analysis. An additive score of ≥ 3 points implies a probability of 81.3% for finding a MIB-1 labeling index of ≥ 5% in the neuropathological analysis of sporadic spinal meningiomas.

### MAC-Spinal Meningioma score and perioperative clinical implications

**Figure 6:**
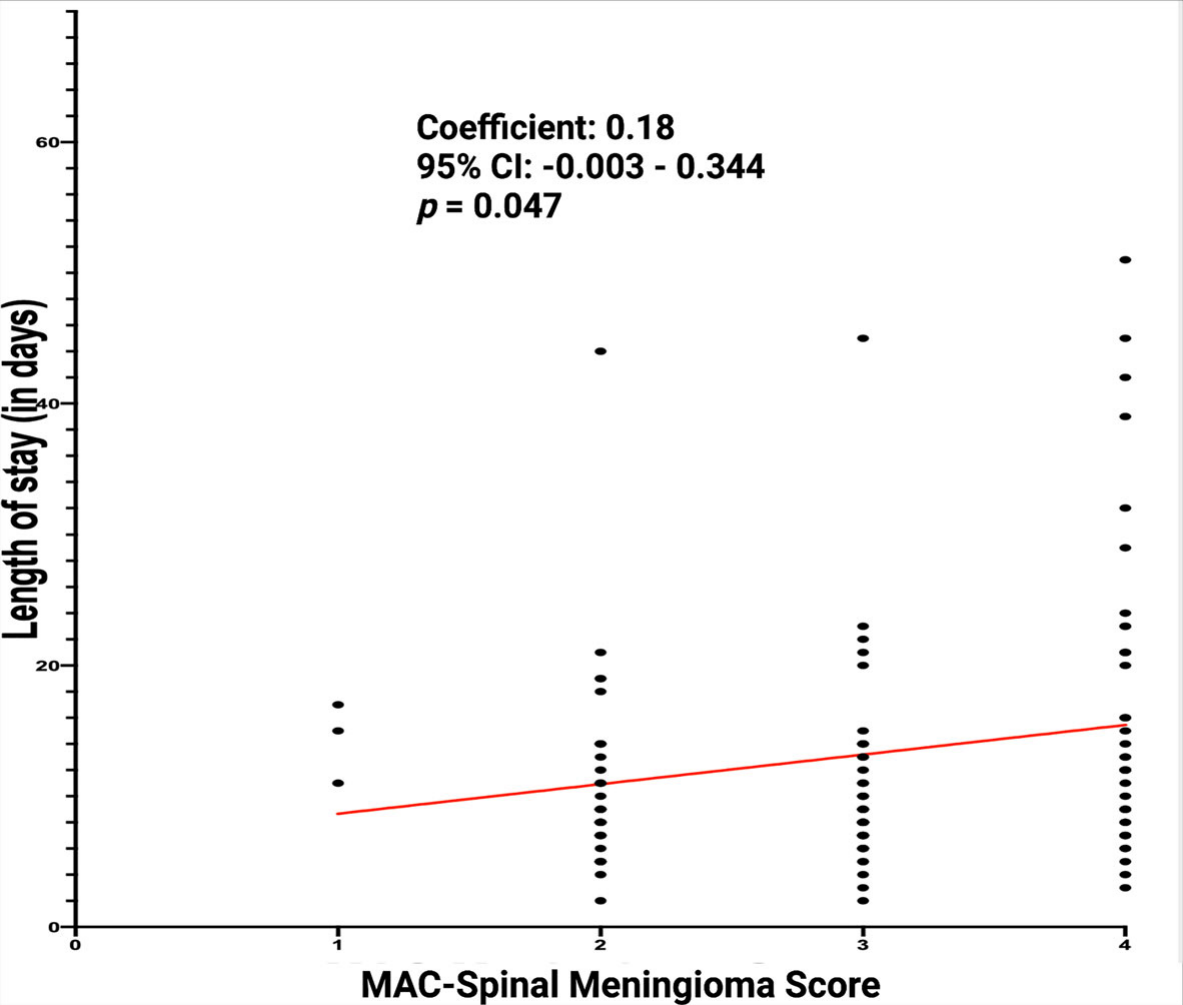
Length of stay (in days) in relation to the MAC-Spinal Meningioma Score of 128 primary sporadic spinal meningiomas.

**Figure 7:**
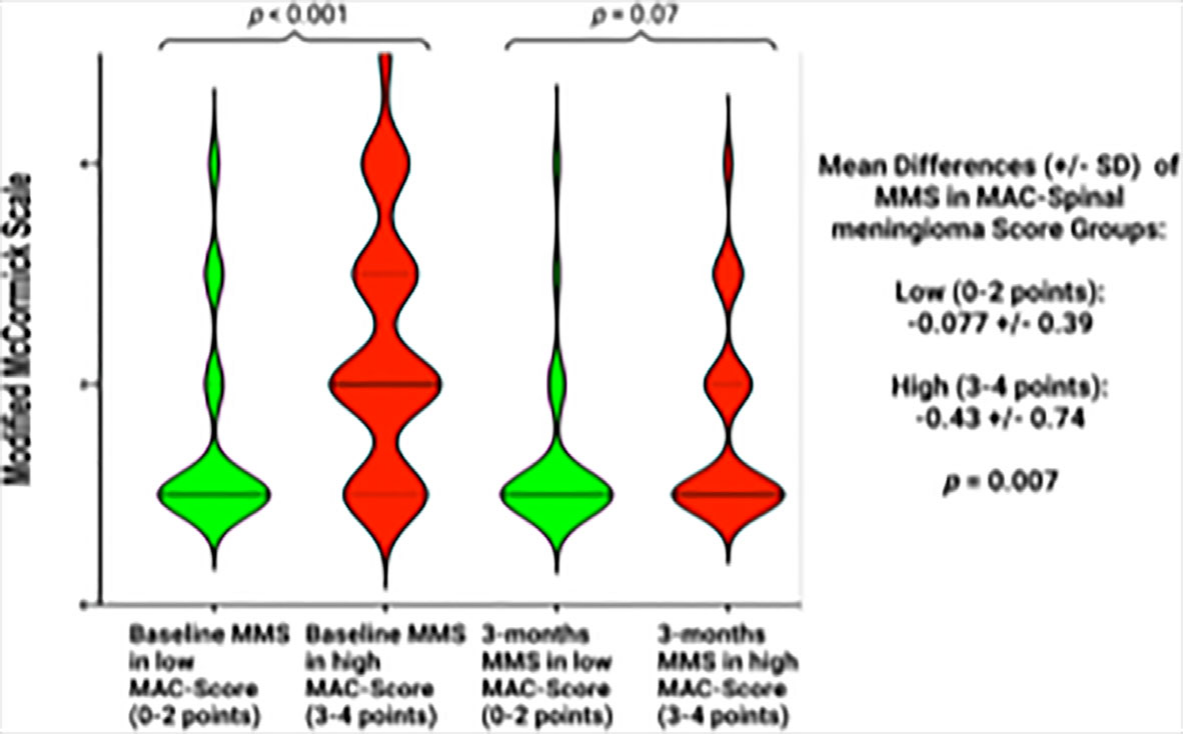
Violin plots displaying the Modified McCormick scale at the preoperative examination, and at 3-months after surgery in patients with a low MAC-Spinal Meningioma score (0-2 points, green violin plot) or a high MAC-Spinal Meningioma score (3-4 points, red violin plot). Violin plots show mean and distribution of Modified McCormick scale. The thick horizontal black lines are the median values. *P*-values of the Student´s *t*-test are reported.

MAC-Spinal Meningioma score was further investigated regarding perioperative clinical implications. The correlation between length of stay (in days) and MAC-Spinal Meningioma score was analyzed. The mean (+/- SD) length of stay in the study cohort was 13.2 (+/- 12.5) days. Spearman´s correlation analysis revealed a statistically significant (*p* = 0.047) positive correlation between the length of stay and MAC-Spinal Meningioma score (*r* = 0.18). **Figure 6** displays the correlation analysis. Furthermore, the association between the course of MMS (baseline – 3-months) and MAC-Spinal Meningioma score was investigated. Patients with a high MAC-Spinal Meningioma Score (3-4 points) had a significantly worse mean (+/-SD) baseline MMS at 2.34 +/- 1.12, whereas patients with a low MAC-Spinal Meningioma Score (0-2 points) had a mean (+/-SD) baseline MMS at 1.51 +/- 0.92 (*p* < 0.001). At 3-months after surgery, patients with a high MAC-Spinal Meningioma score improved significantly more regarding ambulatory functioning. Patients with a high MAC-Spinal Meningioma Score (3-4 points) had a mean (+/-SD) MMS of 1.65 +/- 0.87 at 3-months, and patients with a low MAC-Spinal Meningioma Score (0-2 points) had a mean (+/-SD) MMS of 1.30 +/- 0.74, respectively (*p* = 0.07). Hence, the mean difference of MMS (between baseline and 3-months follow-up) was -0.077 +/- 0.39 in patients with a low MAC-Spinal Meningioma Score (0-2 points), and -0.43 +/- 0.74, respectively (*p* = 0.007). **Figure 7** displays the course of MMS in patients with low- and high-MAC-Spinal Meningioma Score.

## Discussion

Established negative predictors of spinal meningioma recurrence are increased MIB-1 labeling indices, arachnoid invasion, and subtotal resection (17, 49, 50). An increased MIB-1 labeling index is inversely correlated with time to tumor progression in SM and has a positive correlation with the grading of meningiomas (14, 31, 51, 52). SM patients predominantly consulate neurosurgeons *via* elective appointments in an outpatient clinic. Hence, it is essential that patients and their relatives are provided with a tailored and extensive consultation. Nevertheless, MIB-1 labeling indices are not available at the preoperative appointments discussing treatment strategies, extent of resection, imaging intervals, and risk-benefit ratios. The present investigation shows a novel scoring sheet to estimate an elevated MIB-1 labeling index. This potential predictive score includes three routinely and easily determinable characteristics to estimate an elevated MIB-1 labeling index. Furthermore, this scoring system might enable a tailored schedule for imaging in patients who prefer a watch-and-wait strategy instead of surgery. For instance, patients with an increased risk profile (e.g., high ASA class) and the absence of a symptomatic spinal meningioma preferring an initial watch-and-wait strategy, might be scheduled for a more stringent follow-up interval in order to not miss a further tumor progression resulting in a neurological deterioration if they have an increased MAC-Spinal Meningioma score (≥3 points) suggesting a potential increased proliferative activity.

Our results are summarized in the following: (1) a cut-off point of the MIB-1 labeling index set at 5% had the most accurate sensitivity and specificity in the discrimination between stable and progressive SM; (2) Baseline Modified McCormick Scale ≥ 2, age ≥ 65 years at diagnosis, and absence of calcification were significantly associated with an elevated (≥5%) MIB-1 labeling index; (3) the presence of at least one variable among Modified McCormick Scale or age ≥ 65 years at diagnosis in combination with the absence of calcification was a strong predictor of an elevated MIB-1 labeling index; (4) high MAC-Spinal Meningioma Score (3-4 points) is strongly associated with a prolonged length of stay; (5) Despite poorer baseline functioning, patients with a high MAC-Spinal Meningioma score (3-4 points) improve significantly more regarding neurological functioning compared to low MAC-Spinal Meningioma Score patients.

ROC curves were constructed in the present study cohort to evaluate the most accurate cut-off point of the MIB-1 labeling in the estimation of SM recurrence. The present investigation revealed a threshold set at ≥5% as the optimal cut-off point. Cut-off points of MIB-1 labeling index are highly debated in the literature and a broad range (2-20%) of optimum thresholds are reported (29). A recent meta-analysis pooling optimum cut-off points of 43 investigations found a cut-off value set a >4% as accurate regarding risk stratification of overall survival and progression-free survival [29]. However, it has to be reminded that the pooling of MIB-1 labeling index regarding the identification of optimum cut-off values might be more appropriate in a setting analyzing spinal and cranial meningiomas separately. Roser et al. (53) revealed that SMs have significantly lower MIB-1 labeling indices compared to intracranial meningiomas. Therefore, the interlaboratory comparison of reported cut-off points is potentially limited by multiple factors. For instance, the extent of resection has to be considered regarding the specimen sampling because a partially resected tumor tissue implies the risk that the “hotspot” area of maximum proliferative potential is not within the specimen (54). Moreover, it has to be considered that the comparison of interlaboratory MIB-1 labeling indices is also limited by different neuropathological methods (e.g., manual or digital) to determine the MIB-1 labeling index (55).

The results of the multivariable analysis demonstrated that increased baseline MMS ≥2, age ≥ 65 years at diagnosis, and absence of calcification in baseline CT imaging are both significant and independent predictors of an elevated MIB-1 labeling index (≥5%) in SM.

Baseline modified McCormick scale displaying the ambulatory mobility of the patients and their functioning at diagnosis was independently associated with an increased MIB-1 labeling index. To date, this finding has not been described in previous clinicopathological investigations of SMs. However, we have also recently showed in a retrospective institutional series of frontal skull base meningiomas that increased MIB-1 labeling indices are strongly associated with the development or aggravation of new cranial nerve deficits after surgery (45). Moreover, a recent retrospective series evaluating 384 patients who underwent surgery for supratentorial meningiomas revealed that increased MIB-1 labeling indices are significantly associated with Engel class outcomes displaying the postoperative seizure burden (56). Hence, MIB-1 labeling indices might be a potential marker for location-specific symptoms of meningiomas. Furthermore, MIB-1 labeling index has been identified in vestibular schwannomas as diagnostic staining marker which is inversely correlated with the degree of baseline symptoms, duration of symptoms at diagnosis, and postoperative facial nerve function (25, 57). We suggest that those primary sporadic SMs having an increased MIB-1 labeling index grew in a shorter time compared to those with lower MIB-1 labeling indices. Nevertheless, we could not identify differences regarding the tumor size and myelomalacia signs in T2-weighted MR scans among the low or high MIB-1 labeling indices groups. MIB-1 labeling index is known to correlate with the growth rate of primary untreated meningiomas as well as the regrowth of surgically treated meningiomas (58–60).

The present study also showed a simple association between age ≥ 65 years at diagnosis and elevated MIB-1 labeling indices in SM. This relationship was observed in several investigations (61–64). Elderly patients having higher MIB-1 indices were also found by a previous investigation analyzing a prospective database including 1372 cranial meningioma patients (65). Nevertheless, this finding is still controversially debated in the literature. There are also studies which found that proliferation reflected by MIB-1 and progesterone receptor status are not age dependent (66). Furthermore, Maiuri et al. (14) performed a retrospective series of 120 SM patients and dichotomized the study cohort into two groups aged younger or older than 50 years. However, the cut-off set in the mentioned investigation might have been chosen to low because several studies reported mean ages at diagnosis ranging between 60 and 65 years in SM patients (13, 67, 68).

The presence of a calcified spinal meningioma in CT-imaging studies was significantly linked to decreased MIB-1 labeling indices (<5%). Calcification can be observed in up to 25% of meningiomas and was already previously found to be associated with slow growth and lower grade histopathology in cranial meningiomas (69–72). A meta-analysis investigating 777 cranial meningioma patients revealed that tumor calcification is inversely correlated with the meningioma growth rate (69). Moreover, the correlation of CT-imaging signs such as calcification with the immunohistochemical variable MIB-1 labeling index was also investigated in a retrospective series investigating 342 consecutive meningioma patients. Logistic regression analysis of the mentioned study also demonstrated that the absence of calcification is significantly associated with increased MIB-1 labeling indices (47). The implications of calcified or noncalcified meningiomas in terms of a watch and wait approach was also analyzed in a previous series (71). For instance, Rubin et al. (70) followed up both 33 calcified meningioma patients and 27 noncalcified meningioma patients for a mean follow-up time of 65 months. Eighteen of the noncalcified meningiomas showed a tumor growth, whereas only 3 patients of the calcified meningioma group had a meningioma growth. The presence of calcification in SM is more uncommon compared to cranial meningiomas. Gross calcification is described for only 1-5% of SM (73). Previous investigations of calcified SM were predominantly focused on the surgical implications in this rare subgroup of SM regarding functional outcome. Calcified SMs are suggested to be more adherent to spinal nerves and the surrounding layers involving the dura. This condition might be induced by the deposition of calcium in calcified SMs. Several retrospective series debated that the calcification of SMs is strongly associated with poor functional outcomes (4, 74).

The present MAC-Spinal Meningioma score represents a newly created scoring sheet which facilitates the estimation of an increased MIB-1 labeling index in SM. The scoring system might support the preoperative therapy planning and aid physicians in the preoperative consultation with both patients and their relatives because neuropathological characteristics are not available in this setting. Furthermore, SM patients with an elevated MAC-Spinal Meningioma score (≥3) who favor a watch-and-wait policy of their asymptomatic spinal meningiomas should be advised about the need to perform a more stringent schedule of follow-up images. Hence, the MAC-Spinal Meningioma score might facilitate a tailored treatment strategy planning in the preoperative setting. Furthermore, MIB-1 labeling index was demonstrated to be a reliable marker for the time to tumor progression in a prospective trial. This mentioned study analyzed the rates of tumor recurrence and the time to regrowth in WHO grade 1-3 meningiomas. Patients with a MIB-1 index ≥ 5% suffered significantly more often from a tumor progression within the first 24 months after surgery compared to patients with a MIB-1 index ranging between 0 and 4% (75). Moreover, a retrospective series analyzing 239 WHO grade 1 meningiomas showed that the recurrence rates of patients who underwent a gross total resection of a meningioma with a MIB-1 labeling index > 4.5 are similar to patients who had a subtotal resection (60). In a recent institutional intraindividual study of cranial WHO grade 1 and 2 meningiomas we have also confirmed that the MIB-1 labeling indices have a high intraindividual reproducibility which also favors the diagnostic value of the MIB-1 labeling index in terms of tumor progression (76). Therefore, this knowledge might inform the postoperative risk stratification of a meningioma recurrence and facilitate an individualized stringent follow-up strategy. Against this backdrop, it is essential to preoperatively discuss the risk of an increased MIB-1 index and the potential consequences regarding individualized follow-up strategies after surgery. Intraoperative determination of the MIB-1 labeling index to aid the surgical decision making has not been established yet (77, 78). Hence, this scoring system might facilitate the preoperative medical decision-making regarding extent of resection because calcified SMs might be of more benign character, and they are suggested to be associated with poorer functional outcome. Furthermore, the scoring system was also found to be associated with the perioperative course and postoperative course of neurological functioning. A high MAC-Spinal Meningioma score was significantly associated with a prolonged length of stay in the hospital. This strong association might be caused by the fact that patients with a high MAC-Spinal Meningioma score are older and have a poorer baseline MMS. However, we identified that those patients with a high MAC-Spinal Meningioma score improved significantly more regarding neurological functioning compared to those with a low MAC-Spinal Meningioma score. Hence, both groups had no differences in the MMS at 3-months after surgery. This finding might be caused by the fact that those patients with a high MAC-Spinal Meningioma Score had significantly higher MIB-1 labeling indices which suggests that those spinal meningiomas grew in a shorter time and might have not already resulted in a chronic compression of the spinal cord. Hence, those patients might have a better spinal plasticity. All in all, spinal meningioma patients with a high MAC-Spinal Meningioma score might be educated about a potentially faster growing meningioma with an increased MIB-1 labeling index, and a longer length of stay in the hospital. Nevertheless, those patients with a high MAC-Spinal Meningioma Score have surprisingly a nearly identical ambulatory functioning at 3-months after surgery. Consequently, surgical treatment for patients with a high MAC-Spinal Meningioma Score might be highly recommended due to the following reasons: 1) Prevention of the risk of further tumor progression potentially resulting in further neurological deterioration; 2) despite poorer baseline functioning, excellent chances to recover and achieve a nearly identical neurological functioning as patients with a low MAC-Spinal Meningioma Score at 3-months after surgery.

The present investigation has several limitations. Despite the data were acquired from a highly selective and homogeneous cohort, the retrospective design suffered from the potential limitations of a single institutional series. Additionally, MIB-1 labeling indices have to be interpreted with caution due to potential interobserver variability. Several approaches are possible to determine the MIB-1 labeling index, and digital imaging analysis systems might provide a more objective method because it enables a greater number of microscopic fields for the analysis (79). Furthermore, a multicentric prospective trial including a homogeneous study cohort and detailed data has to provide an external validation of the newly created MAC-Spinal Meningioma scoring proposal for sporadic spinal meningiomas.

## Conclusion

MIB-1 labeling index seems to be strongly correlated with an increased risk of tumor progression in sporadic spinal meningioma. The present investigation provides a proposal for a novel scoring sheet (“MAC-Spinal Meningioma”), which might facilitate the preoperative estimation of the MIB-1 labeling index. Moreover, this scoring system might enhance the preoperative surgical decision-making process and guide a tailored treatment strategy in terms of risk-benefit analysis.

## Data availability statement

The raw data supporting the conclusions of this article will be made available by the authors, without undue reservation.

## Ethics statement

The studies involving human participants were reviewed and approved by Ethics committee of University Hospital Bonn. Written informed consent for participation was not required for this study in accordance with the national legislation and the institutional requirements.

## Author contributions

Data acquisition was performed by JW; JW, MH, and EG performed the data interpretation. Writing and creation of figures were performed by JW, MH, and EG. Proof reading was done by AG, TL, FS, AB, UH, HV, and EG. All authors contributed to the article and approved the submitted version.

## Acknowledgments

The graphical abstract in this article was created using BioRender.

## Conflict of interest

The authors declare that the research was conducted in the absence of any commercial or financial relationships that could be construed as a potential conflict of interest.

## Publisher’s note

All claims expressed in this article are solely those of the authors and do not necessarily represent those of their affiliated organizations, or those of the publisher, the editors and the reviewers. Any product that may be evaluated in this article, or claim that may be made by its manufacturer, is not guaranteed or endorsed by the publisher.
