# Supplementary figures and images for: MAC-spinal meningioma score: A proposal for a quick-to-use scoring sheet of the MIB-1 index in sporadic spinal meningiomas
Source: Front Oncol. 2022 Aug 26;12:966581. doi: 10.3389/fonc.2022.966581 (PMC9459241; doi:10.3389/fonc.2022.966581)

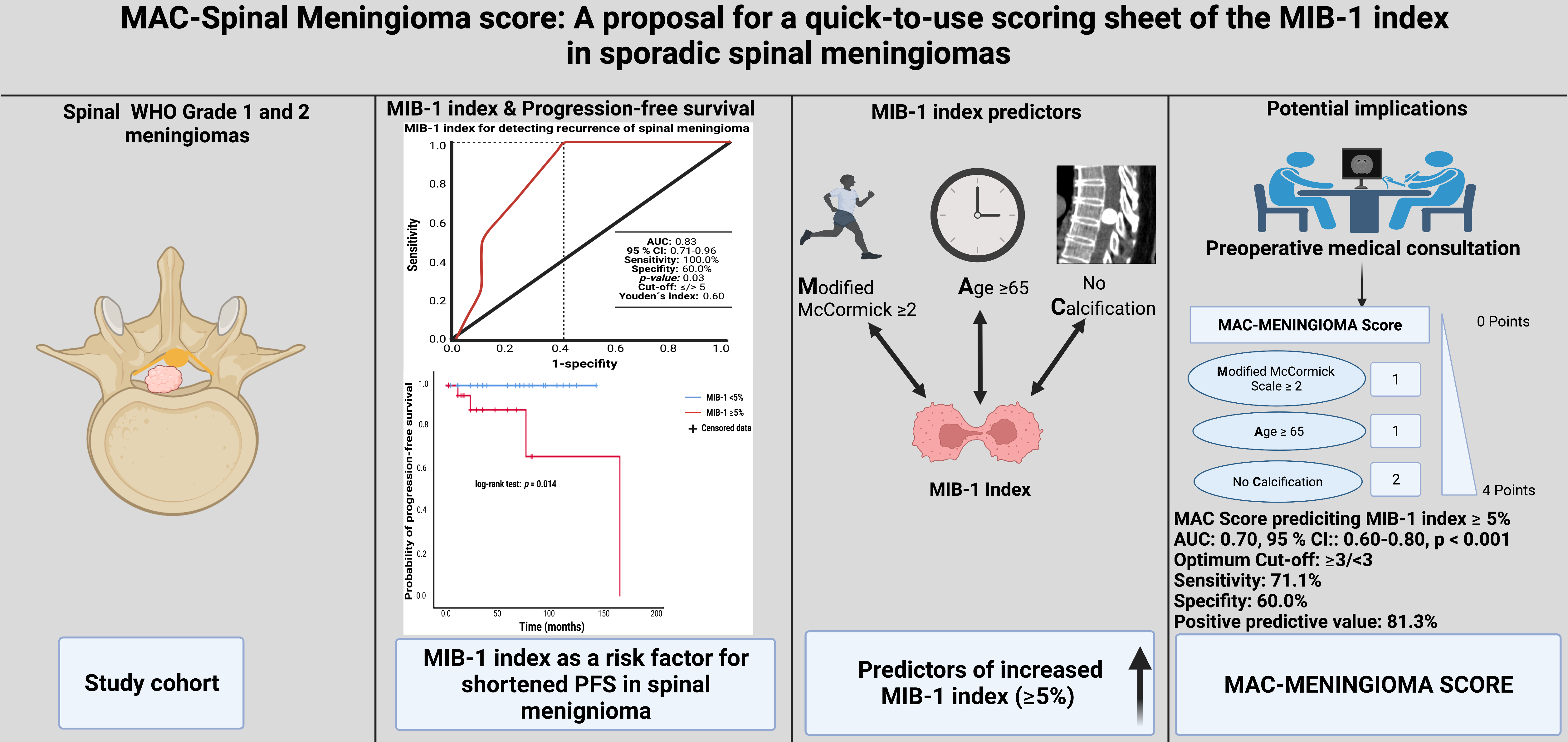

Supplement: Supplementary file 1 [file Image_1.tif]
